# Validation of analytical methods in GMP: the disposable Fast Read 102® device, an alternative practical approach for cell counting

**DOI:** 10.1186/1479-5876-10-112

**Published:** 2012-05-31

**Authors:** Monica Gunetti, Sara Castiglia, Deborah Rustichelli, Katia Mareschi, Fiorella Sanavio, Michela Muraro, Elena Signorino, Laura Castello, Ivana Ferrero, Franca Fagioli

**Affiliations:** 1 Paediatric Onco-Haematology, Stem Cell Transplantation and Cellular Therapy Division, Regina Margherita Children’s Hospital, Piazza Polonia 94, Turin, 10126, Italy; 2 Department of Paediatrics, University of Turin, Turin, Italy

**Keywords:** Cell count, Cell factory, Cell therapy, Validation methods, GMP

## Abstract

**Background:**

The quality and safety of advanced therapy products must be maintained throughout their production and quality control cycle to ensure their final use in patients. We validated the cell count method according to the International Conference on Harmonization of Technical Requirements for Registration of Pharmaceuticals for Human Use and European Pharmacopoeia, considering the tests’ accuracy, precision, repeatability, linearity and range.

**Methods:**

As the cell count is a potency test, we checked accuracy, precision, and linearity, according to ICH Q2. Briefly our experimental approach was first to evaluate the accuracy of Fast Read 102® compared to the Bürker chamber. Once the accuracy of the alternative method was demonstrated, we checked the precision and linearity test only using Fast Read 102®. The data were statistically analyzed by average, standard deviation and coefficient of variation percentages *inter* and *intra* operator.

**Results:**

All the tests performed met the established acceptance criteria of a coefficient of variation of less than ten percent. For the cell count, the precision reached by each operator had a coefficient of variation of less than ten percent (total cells) and under five percent (viable cells). The best range of dilution, to obtain a slope line value very similar to 1, was between 1:8 and 1:128.

**Conclusions:**

Our data demonstrated that the Fast Read 102® count method is accurate, precise and ensures the linearity of the results obtained in a range of cell dilution. Under our standard method procedures, this assay may thus be considered a good quality control method for the cell count as a batch release quality control test. Moreover, the Fast Read 102® chamber is a plastic, disposable device that allows a number of samples to be counted in the same chamber. Last but not least, it overcomes the problem of chamber washing after use and so allows a cell count in a clean environment such as that in a Cell Factory. In a good manufacturing practice setting the disposable cell counting devices will allow a single use of the count chamber they can then be thrown away, thus avoiding the waste disposal of vital dye (e.g. Trypan Blue) or lysing solution (e.g. Tuerk solution).

## Background

The use of human cell based medical products (hCBMPs) in a patient-specific manner for cell therapy purposes raises specific issues pertaining to quality control testing designs for each product under examination [1]. European Community (EC) Directive 2001/83/EC relating to medicinal products for human use defines a hCBMP as a medicinal product which has properties to treat or prevent disease in human beings. Regulations and guidelines for CBMP production follow those of conventional medicinal products [2-4]. The European Parliament Regulation N. 1394/2007 on advanced therapy medicinal products, amending the 2001/83/EC Directive, completed the regulatory setting on advanced therapies to be used in Member States [5]. The manufacturing process of CBMPs has to comply with the principles and guidelines of good manufacturing practice (GMP) for medicinal products for human use published by the European Commission [6,7]. GMP ensures that products are consistently produced and controlled to the quality standards required for their intended use, from the collection and manipulation of raw materials to the processing of intermediate products, the quality controls, storage, labelling and packaging, and release. In general, when a CBMP enters the clinical development phase, the same principles as those for other medicinal products apply [8]. There should be a careful design and validation of the entire manufacturing process of CBMPs, including cell harvesting, cell manipulation processes, the maximum number of cell passages, and combinations with other components of the product, filling, packaging, etc. In order to ensure product safety and efficacy, each step of the manufacturing process of active substances and supportive components should all be demonstrated, as should be the control of the final product. The quality and safety of the cell preparations should be ensured by implementing a quality system that guarantees the certification and the traceability of every batch of material and supply utilized for the procedures and the correct utilization and cleaning of instruments and locations necessary for stem cell manipulation. Furthermore, the organization structure, qualification and training status of the personnel, and the appropriate equipment, should also comply with current GMP standards. An important aspect of advanced therapies is the necessity to process CBMPs in an aseptic environment, to avoid terminal sterilization which would lead to damage to, and the ineffectiveness of, the cell product. Each manufacturing operation requires an appropriate environmental cleanliness level in the operational state to minimize the risks of particulate or microbial contamination of the product or materials being handled. The application of the GMPs for aseptic production, besides checking all the aspects related to the process, is aimed at minimizing possible contamination factors (personnel, environment, equipment, manufacturing and storage conditions) to ensure the safety of the final product. The quality and safety of advanced therapy products must be maintained throughout their production and quality control (QC) cycle, thus ensuring their final use in the patient. An extensive characterization of the cell therapy product (CTP) should be established in terms of identity, purity, potency and suitability for their intended use. In this context, the cell count, that will indicate the CTP dose, is a potency test. On these bases we validated the cell count method according to the International Conference on Harmonization (ICH) Q2 Guidelines [9] and European Pharmacopoeia (EP) [10], taking into account the tests’ accuracy, precision, repeatability, linearity and range. According to the ICH Q2 [9]: “*accuracy* expresses the closeness of agreement between the value which is accepted as either a conventional true value or as an accepted reference value and the value found; *precision* of an analytical procedure expresses the closeness of agreement (degree of scatter) between a series of measurements obtained from the multiple sampling of the same homogeneous sample under the prescribed conditions; *repeatability* (also termed *intra*-assay precision) expresses the precision under the same operating conditions over a short interval of time; *linearity* of an analytical procedure is its ability (within a given *range*) to obtain test results which are directly proportional to the concentration of an analyte in the same sample”. There are various manual or automatic, cell count methods. Among the manual cell count methods, the Bürker chamber is seen as the reference method and is the only one described in EP [10]. Alternative manual cell counting chambers include the following: Malazzes, Thoma, Lemaur, Nageotte, Neubauer, Neubauer impaired, Agasse Lafont, and Fuch-Rosenthal. These devices basically differ in the type of ruling that they also have cover glasses of different sizes for the counting chamber. All these double net rulings are made of glass and are not disposable. There are also disposable cell count devices as Kovas slides and Fast Read 102 ® slides. In a GMP setting the latter devices will allow the count chamber to be used only once and then be trashed so that the disposal of waste of vital dye (e.g. Trypan Blue) or lysing solution (e.g. Tuerk solution) can be avoided. Moreover, although some automatic counters are marketed, for GMP settings, their associated software should comply with 21 CFR Part 11 [11-14]. On these bases, our primary aim was to validate a disposable cell count method in GMP conditions, to be included in the Validation Master Plan (VMP) to be submitted to the Regulators, to obtain accreditation of our Cell Factory to produce CTPs. We decided to use a manual cell count and validate, according to GMP rules, a disposable device, Fast Read 102 ®, already used in P3 laboratory by our group.

We therefore sought to evaluate whether Fast Read 102 ® could substitute the Bürker chamber in terms of accurancy. Once this hypothesis was proved, we tested repeatability, linearity and range only using Fast Read 102 ®. Two cell subpopulations were chosen for the validation procedure: mononuclear cells (MNCs), as a prototype of lymphocytes, and mesenchymal stem cells (MSCs), both cell therapy products (CTP) that we will produce for immunotherapy and regenerative medicine. In these settings, we validated viability by Trypan Blue dye. We isolated MNCs and MSCs from whole peripheral blood (wPB) and whole bone marrow (wBM) respectively. We also validated the wPB and wBM cell counts, using Tuerk dye as a red cell lysing solution, to establish the best range of a sample dilution. Finally, the outcome of this experiment served to test whether, having found the dilution range, it might also be suitable for the MNC and MSC counts.

## Methods

**Figure 1:**
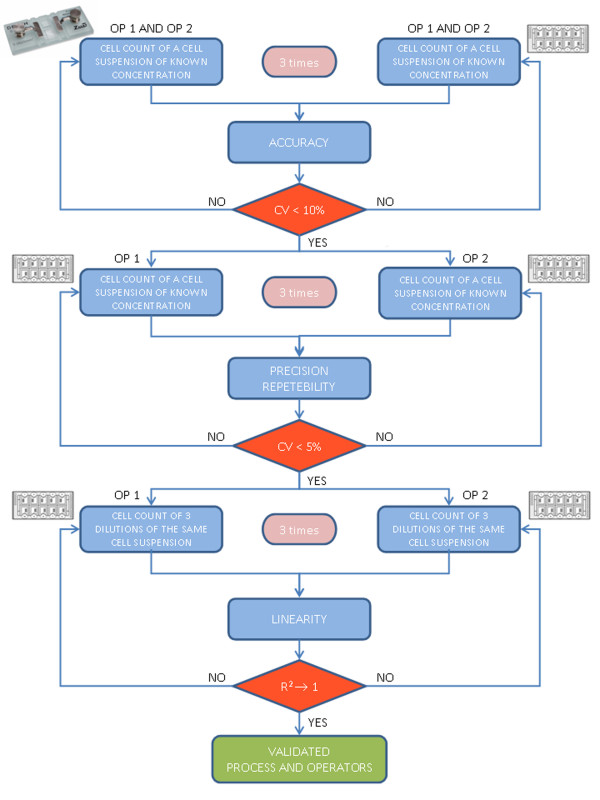
**Cell count validation protocol. **According to ICH Q2, the test was performed three times under the same operating conditions by Op1 and Op2. The concentration of the cell suspension was previously quantified using the Bürker chamber. Both operators then evaluated the accuracy of the method comparing the Bürker chamber with the Fast Read 102® chamber, performing a total (non-viable) cell count. In order to evaluate the precision and the repeatability of the method, *intra *and *inter *operator CV% was calculated using a viable cell count by Trypan Blue vital dye. The acceptance criteria were: *inter *and *intra *operator CV% < 10 % (total cell count); *intra *and *inter *operator CV% < 5 % (viable cell count). For linearity, we tested serial dilutions, in the following range: undiluted, 1:2, 1:4, 1:8, 1:16, 1:32, 1:64, 1:128. Op1 and Op2 performed a total cell count three times for each dilution. On the basis of the results, the regression line was calculated and the optimal range of dilutions was determined.

The cell count validation protocol was performed as shown in the flow chart (Figure 1). We used two different cell products, MNCs and MSCs. The validation procedure was performed by two operators (Op): Op1 and Op2.

### MNC isolation

PB, obtained from healthy donors after informed consent, was layered on a Hystopaque (Sigma Aldrich, Milan, Italy) gradient (1.077 g/ml density). The cells were centrifuged at 400 g for 30 minutes. The cells in the interphase were collected, washed twice with phosphate buffered saline (PBS) 1X (200 g for 10 minutes) and resuspended in CellGrow® SCGM Medium (Cell Genix, Freiburg Germany) containing 5% of Human Sera Type AB (Lonza, Verviers, Belgium).

### BM MSC isolation and expansion

Whole Bone Marrow (wBM) MSCs were isolated from human BM obtained by aspiration from the posterior iliac crest of healthy donors after written informed consent. The MSC frequency in BM was about 1/10^4^ cells [15]. Briefly, wBM was seeded at a density of 100,000/cm^2^ in MesenCult® Proliferation Kit (MCPK) (Stemcell Technologies, Vancouver, BC, Canada) in 75 or 150 cm^2^ T-flasks and maintained at 37°C with a 5% CO_2_ atmosphere. After 5 days, the non-adherent cells were removed and re-feeded every three to four days. At confluence, they were detached, and re-plated at different densities for one to four passages [16]. To perform the cell count procedure, at the second-fourth passages of culture (P2-P4), the MSCs were detached and resuspended in MCPK.

### Bürke*r* cell count

**Figure 2:**
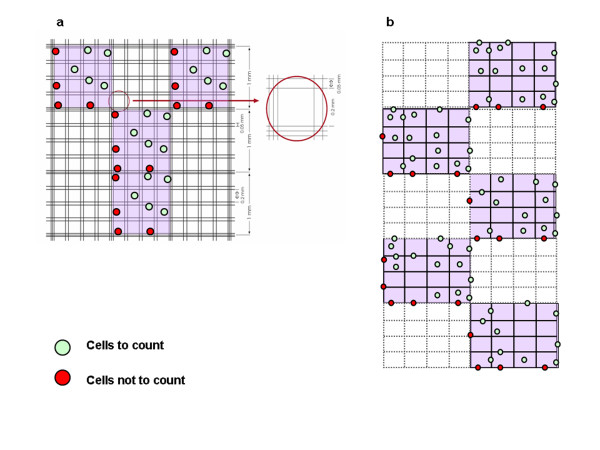
**Bürker chamber and Fast Read 102® cell count method. **The Bürker chamber has 9 large squares (1 mm^2^ each), divided by double lines (0.05 mm apart) into 16 group squares. The double lines form small 0.0025 mm^2^ squares. The Chamber depth is 0.1 mm. The cells were counted in each of the 4 large squares (identified by the triple line and shaded in the figure). At the end of the procedure the operators calculate the average of the 4 readings (from 4 large squares) and calculate the cell concentration as follows:. $\left[\frac{\text{Cell}}{ml}\right]=\left(\frac{\sum \text{Cells}\;\text{counted}\;in\;4\;\text{squares}}{4}\right)\times \text{dilution}\;\text{factor}\times 10^{4}$. Fast Read 102® chamber, a plastic device with a slide divided into 10 chambers. Each chamber contains a grid with 10 squares, subdivided into 16 small squares. When the chamber was filled, the cells distributed in the 5 squares (black lines) were counted, taking into consideration, for each chamber, a size of 1 x 1 mm, a depth of 0.1 mm and a volume of 0.1 μl per square, the cell concentration (cells/ml) was determined by the formula: $\left[\frac{\text{Cell}}{ml}\right]=\left(\frac{\sum \text{cells}\;\text{counted}\;in\;5\;\text{squares}}{5}\right)\times \text{dilution}\;\text{factor}\times 10^{4}$

The Bürker chamber (Figure 2A) has 9 large squares (1 mm^2^ each), divided by double lines (0.05 mm apart) into 16 group squares. The double lines form small 0.0025 mm^2^ squares. The Chamber depth is 0.1 mm. The cells were counted as reported in Figure 2A. Briefly, both operators take 10 μl of cell suspension with a micropipette and put them in the cell count chamber and then count the cells in each of the 4 large squares (identified by the triple line and shaded in the figure). At the end of the procedure the operators calculate the average of the 4 readings (from 4 large squares) and calculate the cell concentration as follows:

(1) $$\left[\frac{Cell}{ml}\right]=\left[\frac{\sum cell\;counted\;in\;4\;large\;squares}{4}\right]\times \left(dilution\;factor\right)\times 1\times 10^{4}$$

### Fast Read 102® cell count

The Bürker chamber cell count was compared to the Fast Read 102® count. The Fast Read 102® is a plastic slide, with 10 chambers, that allows single-use, quickly and effectively, and the simultaneous reading of 10 cell counts. The operators transferred 10 μl of cell suspension to be analysed into each chamber and by capillary action the cell suspension filled the corresponding chamber, allowing a quick read with a microscope. Depending on the focus adjustment of the microscope, the cells can be counted and seen as reflective spheres. The cells were counted as reported in Figure 2B. Briefly, both operators counted the cells in 5 large squares, and calculated the average of the counts to reduce the margin of error. In each large square, they counted all the cells contained in the 16 small squares, including the internal dividing lines. Thus, for each chamber (square size 1 x 1 mm, square depth 0.1 mm and square volume 0.1 μl), the cell count per ml was performed using the following formula:

(2) $$\left[\frac{\text{Cells}}{ml}\right]=\left[\frac{\sum \text{cells}\;\text{counted}\;in\;5\;\text{squares}}{5}\right]\times \left(\text{dilution factor}\right)\times 1\times 10^{4}\;$$

### Accuracy

Accuracy was checked, as above reported*,* according to ICH Q2 [9]. The test was performed on MNCs, by Ops1 and 2, three times under the same operating conditions. The concentration of the cell suspension was previously quantified using the Bürker chamber. To evaluate accuracy, the results obtained with the Bürker chamber were compared to those obtained by using the Fast Read 102® chamber. As for accuracy test, the Ops had to count repeated on the same day with the same sample, to avoid the possibility that the time had affected the cell viability, in this first step of experiments, the accuracy test was performed on total cells without considering cell viability.

On the basis of the accuracy test, after having validated the overlapping of cell count data by the two above described methods, we decided to use the disposable Fast Read 102® chamber, instead of the Bürker chamber, for all subsequent tests.

### Precision and repeatability

On the bases of accuracy results, the precision and repeatability were assayed, according to ICH Q2 [9], using the Fast Read 102® chamber. The test was performed on MNCs and MSCs, by Ops1 and 2, three times under the same operating conditions. Then, in all the cell counts we evaluated cell viability, using Trypan Blue vital dye.

### Viability cell counting by Trypan Blue dye

For viable cell counting the cell suspension was diluted 1:2 by Trypan Blue dye. Briefly, the Ops take, by micropipette, 10 μl of cell suspension and diluted it, by pipetting, with an equal volume of Trypan Blue dye. They then transferred 10 μl of this diluted cell suspension, to be analysed, into each chamber of the Fast Read 102® slides.

### Linearity and range on wPB and wBM count

In order to test these two analytical methods, as we had isolated MNCs and MSCs cells from wPB and wBM respectively, first we decided to check linearity and range, using Fast Read 102®, on these viscous tissues highly enriched in red blood cells (RBC). For this purpose, according to ICH Q2 [9], Ops used serial diluitions in the following ranges: undiluted, 1:2, 1:4, 1:8, 1:16, 1:32, 1:64, 1:128. We then diluted 10 μl of wPB and wBM with different volumes of Tuerk lysing (Fluka), an acetic acid and gentian violet solution, that haemolyzes RBCs and stains the nuclei of white blood cells (WBC) blue. To avoid any potential Tuerk lysing solution or Trypan Blue vital dye interference, the test only considers total cell count and not cell viability. The Ops performed the cell count three times for each dilution. On the basis of the results, the regression line was calculated and the optimal range of dilutions was determined.

### Linearity and range on MNCs and MSCs

According to ICH Q2 [9], as above described, we also tested linearity and range on MNCs and MSCs using serial dilutions, in the following ranges: undiluted, 1:2, 1:4, 1:8, 1:16, 1:32, 1:64, 1:128. Ops 1 and 2 performed the cell count three times for each dilution. The test was performed without considering cell viability to be compared to previous described data on wPB and wBM. On the basis of the results, the regression line was calculated and the optimal range of dilutions was determined.

### Statistical analysis

The statistical analysis was performed by calculating average, standard deviation (SD) and *inter* and *intra* operator coefficients of variation (CV%).

The accuracy of the method was determined by calculating *inter* and *intra* operator CV% (Bürker vs Fast Read 102® chamber); similarly, the precision and the repeatability of the method were established by evaluating the *inter* and *intra* operator CV%.

The acceptance criteria for accuracy was, as shown in the flow chart in Figure 1, CV% < 10 % (total cell count). For precision and repeatability, the acceptance criteria were, as shown in the flow chart in Figure 1, CV% < 5% (viable cell count). The regression line was calculated for linearity and the range test.

### Statement of ethical approval

Bone Marrow (BM) and peripheral blood (PB) were obtained from healthy donors after written informed consent in accordance with the approval of the Ethics Committees, of the Regina Margherita, S.Anna and Mauriziano hospitals, and in compliance with the Helsinki Declaration.

## Results and discussion

Our primary aims were to validate, in a GMP conditions, a disposable cell count method to be included in a Validation Master Plan (VMP) to be submitted to the Regulators, in order to obtain the accreditation of our Cell Factory to produce CTPs. In this context the cell count is a potency test, according to EP, which define the cell dose during *ex-vivo* expansion and batch release. In a GMP setting the cell counting using disposable devices is a very important starting point as it will allow the single use of the count chamber and thus avoid disposal of vital dye (e.g. Trypan Blue) or lysing solution (e.g. Tuerk solution). As we had experience on cell counting by Fast Read 102 ® slides (despite there being other disposable cell count chambers availble), we first tested whether Fast Read 102 ® devices might substitute Bürker chamber in terms of accuracy. Once this hypothesis was proved, we tested repeatability, linearity and range only using Fast Read 102 ®. Two cell subpopulations were chosen for the validation procedure: MNCs, as a subset of lymphocytes, and MSCs: both CTPs that we will produce for immunotherapy and regenerative medicine.

To minimize the error due to the diverse pipetting procedures, the operators standardized this critical issue. This practice was especially necessary because, in order to standardize the procedure, we decided to use forward pipetting for wPB, wBM and for less viscous cell suspensions.

For every test (accuracy, precision, repeatability, linearity and range) the Ops, according to ICH Q2, performed three cell counts, under the same operating conditions.

Three repetitions for each count was sufficient for a statistical analysis and, moreover, the CV% and SD we obtained corroborate, in our opinion, our experimental approach.

On the basis of the accuracy test once we had validated the overlapping of cell count data by both above described methods, we decided to use the disposable Fast Read 102® chamber, instead of the Bürker chamber, for all subsequent tests.

### Accuracy

As accuracy expresses the closeness of agreement between the value, which is accepted as either a conventional true value or as an accepted reference value and the value found, we decided that the use of MNCs was sufficient to validate this point of method.

Ops 1 and 2 performed their cell count three times. First with the Bürker chamber, the standard cell count method described in EP, in order to detect the exact cell suspension concentration, and then with the Fast Read 102® chamber. As already explained above, since this first phase was lengthy, we decided to avoid evaluating cell viability that would have distorted the results.

**Figure 3:**
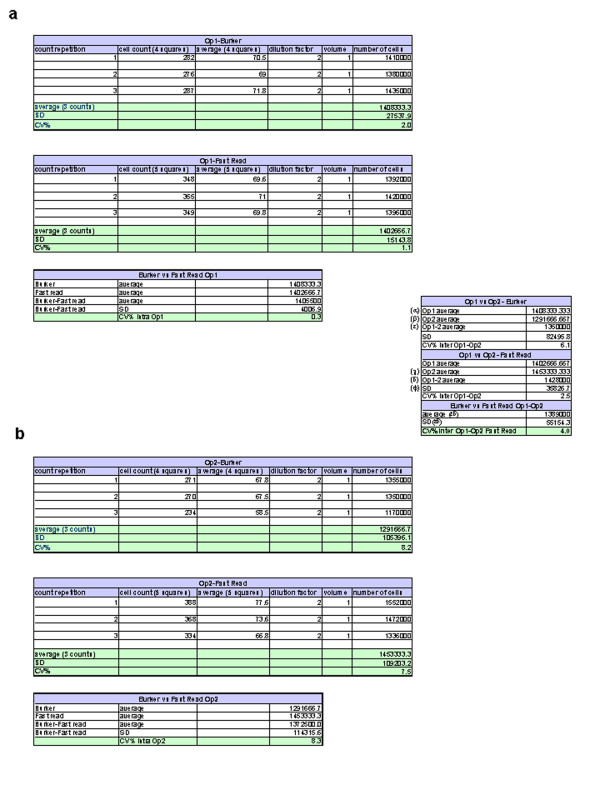
**Accuracy. **Accuracy data demonstrated that the cell count obtained by the disposable Fast Read 102® cell count device is comparable to the Bürker cell count. Each Operator calculated the average and SD of its three counts for both counting devices to obtain the *intra *operator CV%. Op1 and Op2 then calculated *intra * operator CV% between Bürker *vs *Fast Read102® (**a**: Op1 raw data; **b**: Op2 raw data). Finally, *inter *operator CV% (Bürker *vs *Fast Read 102®) was reported **(c).** CV = coefficient of variation, Op1 = operator 1, Op2 = operator 2.

Each operator calculated the average and SD of its three counts for both count devices to obtain the *intra* operator CV%. All the data obtained are summarized in Figure 3. The *intra* operator CV% was <10% [Op1 (Bürker): CV% = 2; Op2 (Bürker): CV% = 8.2; Op1 (Fast Read102®): 1.1; Op2 (Fast Read102®): 7.5]. Op2’s CV% in the accuracy testing was very high compared to Op1’s, but fulfills the established acceptance criteria (< 10%). This CV% appears much lower when testing precision and repeatability probably due to the improvement of the confidence with the method. Ops 1 and 2 then had to calculate *intra* operator CV% (Bürker *vs* Fast Read102®): similarly, the *intra* operator CV% was <10 % [*intra* Op1 CV% (Bürker *vs* Fast Read102®) = 0.3; *intra* Op2 CV% (Bürker *vs* Fast Read102®) = 8.3]. Finally, *inter* operator CV% (Bürker *vs* Fast Read 102®) was calculated and was under 10% [CV% (Op1-Op2 Bürker) = 6.1; CV% (Op1-Op2 Fast Read 102®) = 2.5; CV% (Op1-Op2 Bürker *vs* Fast Read 102®) = 4]. As the accuracy and repeatability criteria were satisfied, Fast Read 102® was considered comparable to Bürker chamber and so the following tests were performed only with the disposable Fast Read 102® chamber.

### Precision and repeatability

**Figure 4:**
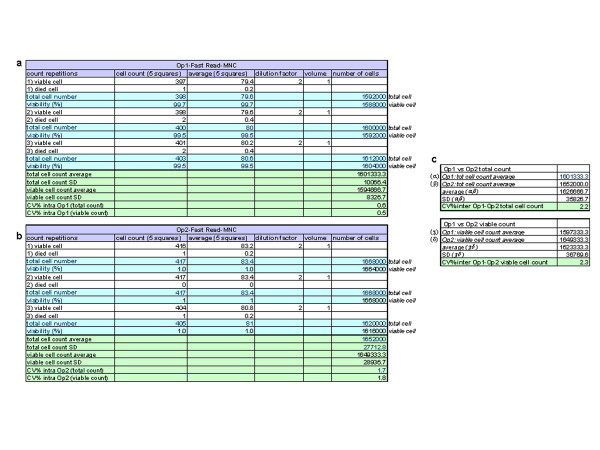
**Precision and Repeatability on MNCs. **On the basis of accuracy data, the following experiments were made to test precision, repeatability, linearity and range only by using Fast Read 102®. The assay was performed only using the Fast Read 102® chamber on MNCs. Operators 1 and 2 tested their cell counts three times. Cell viability was evaluated using Trypan Blue vital dye with a 1:2 dilution. To obtain the *intra *operator CV%, each operator calculated the average and SD of their three counts **(a)**: Op1 raw data; **(b)**: Op2 raw data. The *inter *operator CV% viability was then calculated **(c)**. CV = coefficient of variation, MNC = mononuclear cells, Op1 = operator 1, Op2 = operator 2.

As previously and amply explained above, the test was performed on MNCs and on MSCs using the Fast Read 102® chamber. Op1 and Op2 performed the cell count three times. The cell viability was evaluated using Trypan Blue vital dye, with a 1:2 dilution, as above described. To obtain the *intra* operator CV%, Op1 and Op2 calculated the average and SD of their three counts for each cell type (MNCs and MSCs). All the data are summarized in Figures 4 (MNCs) and 5 (MSCs). For both MNCs and MSCs, the viability *intra* operator CV% was <5% [Op1 (MNCs): CV % = 0.5; Op2 (MNCs): CV% = 1.8 ; Op1 (MSCs): CV % = 4.4; Op2 (MSCs): CV% = 2.7], thus demonstrating the repeatability of operators. Ops 1 and 2 then calculated the viability of *inter* operator CV% [CV% (Op1-Op2 MNCs) = 2.8; CV% (Op1-Op2 MSCs) = 4.2]. These data demonstrated that the method is valid as it is both repeatable and precise.

**Figure 5:**
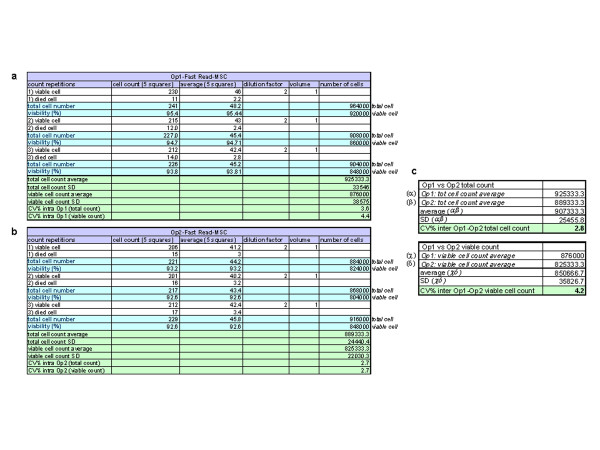
**Precision and Repeatability on MSCs. **As previously reported, also for MSCs, the assay was performed only using the Fast Read 102® chamber on MSCs. Operators 1 and 2 tested their cell counts three times. Cell viability was evaluated using Trypan Blue vital dye with a 1:2 dilution. To obtain the *intra *operator CV%, each operator calculated the average and SD of their three counts (**a:** Op1 raw data; **b:** Op2 raw data). The *inter * operator CV% viability was then calculated **(c).** CV = coefficient of variation, MSC = mesenchymal stem cells, Op1 = operator 1, Op2 = operator 2.

### Linearity and range on wPB and wBM

In order to test these two analytical methods, as we isolated MNCs and MSCs cells from wPB and wBM respectively, first we decided to set up linearity and range, according to ICH Q2, using serial dilution ranges (undiluted ranges: 1:2, 1:4, 1:8, 1:16, 1:32, 1:64, 1:128) of these viscous tissues that are highly enriched in red blood cells (RBC), with the Tuerk lysing solution above described. To avoid any potential interference of the Tuerk lysing solution and the Trypan Blue vital dye, in this case, the test only considers the total cell count and not cell viability. Ops1 and 2 were thus able to verify the best range of dilution to use.

**Figure 6:**
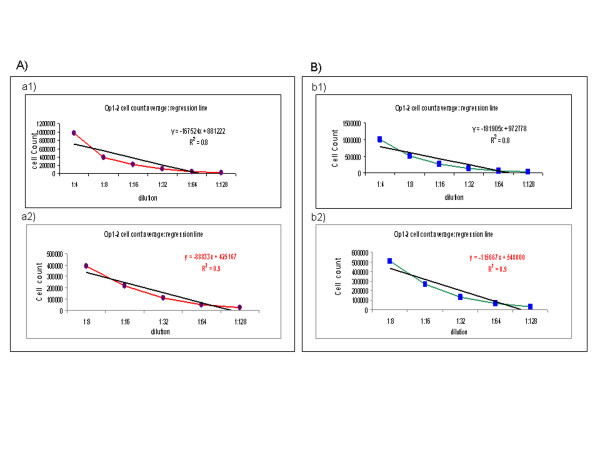
**Linearity and range on wPB and wBM. **Linearity and range was evaluated by Fast Read 102 ® device, as above described. The assay was performed on wPB **(A)** and wBM **(B)**. The Operators tested different dilutions of cell suspension with Tuerk solution (undiluted, 1:2, 1:4, 1:8, 1:16, 1:32, 1:64, 1:128) in order to verify the best range of dilution to use. To avoid any potential interference of the Tuerk lysing solution and the Trypan Blue vital dye, in this case, the test only considers the total cell count and not cell viability. The regression line was calculated using the average of values obtained from Op1 and Op2’s counts at each dilution. The best range of dilution, in order to obtain a slope line value very similar to 1, is between 1:8 and 1:128. a1) wPB dilution range: 1:4, 1:8, 1:16, 1:32, 1:64, 1:128; a2) wPB dilution range: 1:8, 1:16, 1:32, 1:64, 1:128; b1) wBM dilution range: 1:4, 1:8, 1:16, 1:32, 1:64, 1:128; b2) wBM dilution range: 1:8, 1:16, 1:32, 1:64, 1:128. Op1 = operator 1, Op2 = operator 2.

There were three different steps through which it was possible to re-evaluate *intra* and *inter* operator precision and the range of dilutions in which linearity is maintained: 1) *Intra operator precision:* Op1 and Op2 performed three cell counts and calculated the *intra* operator CV% at each dilution for both wPB and wBM. The value of *intra* operator CV% was always <10%; 2) *Inter operator precision:* Op1 and Op 2 calculated the *inter* operator CV% at each dilution for both wPB and wBM. The value of *inter* operator CV% was always <10%; 3) *Regression Line:* calculated by using the average of values obtained from Op 1 and 2’s counts at each dilution. The best dilution range in order to obtain a slope line value very similar to 1, is between 1:8 and 1:128 [R^2^ (wPB) = 0.9; R^2^ (wBM) = 0.9]. All the data are summarized in Figure 6.

### Linearity and range on MNCs and on MSCs

According to ICH Q2 [9], as described above, we tested serial dilutions, in the following range: undiluted, 1:2, 1:4, 1:8, 1:16, 1:32, 1:64, 1:128. Op1 and Op2 performed the cell count three times for each dilution. The test was performed without considering cell viability, as described above, to compare all the “linearity and range” data.

**Figure 7:**
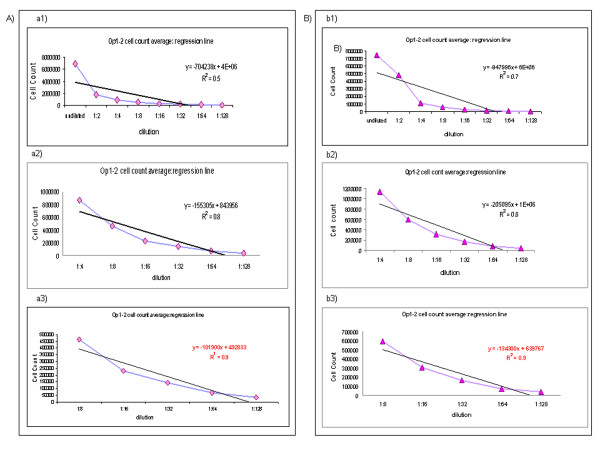
**Linearity and range on MNCs and MSCs. **The assay was performed on MNCs **(A)** and MSCs **(B)**, using Fast Read 102 ®, as previously reported. The Operators tested different dilutions of cell suspension (undiluted, 1:2, 1:4, 1:8, 1:16, 1:32, 1:64, 1:128). The test was performed without considering cell viability, as described above, to compare all the “linearity and range” data. The regression line was calculated by using the average of values obtained from Op1 and Op2’s counts at each dilution. The best range of dilution, in order to obtain a slope line value very similar to 1, is between 1:8 and 1:128. a1) MNCs dilution range: undiluted, 1:2, 1:4, 1:8, 1:16, 1:32, 1:64, 1:128; a2) MNCs dilution range: 1:4, 1:8, 1:16, 1:32, 1:64, 1:128; a3) MNCs dilution range: 1:8, 1:16, 1:32, 1:64, 1:128; b1) MSCs dilution range: undiluted, 1:2, 1:4, 1:8, 1:16, 1:32, 1:64, 1:128; b2) MSCs dilution range: 1:4, 1:8, 1:16, 1:32, 1:64, 1:128; b3) MSCs dilution range: 1:8, 1:16, 1:32, 1:64, 1:128. Op1 = operator 1, Op2 = operator 2.

There were three different steps through which it was possible to re-evaluate *inter* and *intra* operator precision and the dilution ranges in which the linearity is maintained: 1) *intra* operator precision*:* Op1 and Op2 performed three cell counts and calculated the *intra* operator CV% at each dilution for both MNCs and MSCs. The *intra* operator CV% value was always <10%; 2) *inter operator precision:* Op1 and Op 2 calculated the *inter* operator CV% at each dilution for both MNCs and MSCs. The value of *inter* operator CV% was always <10%; 3) *Regression Line:* calculated using the average of values obtained from the Op1 and Op2 cell counts at each dilution. The best range of dilution, in order to obtain a slope line value very similar to 1, is between 1:8 and 1:128 [R^2^ (MNCs) = 0.9; R^2^ (MSCs) = 0.9. All the data are summarized in Figure 7.

## Conclusions

Cellular therapy is an emerging field in medicine. The employment of stem cell types in clinical studies, requires formal approvals by respective regulatory bodies. Such approvals require the manufacturing, processing and testing of cellular products [17-19] according to the current GMP guide lines concerning medical products and investigational medicinal products for human use [1-8]. During CTP manufacturing, every step should be taken to demonstrate their suitability for routine processing and should be validated in order to produce cells of the required quality. All biological products must meet the prescribed requirements of safety, purity and potency and no lot of any licensed product may be released by the manufacturer prior to the completion of tests for the conformity with standards applicable to such products including potency [20]. The cell count procedure is a critical point in CTP production, in both the manufacturing phases and in lot release. Indeed, in the former, the cell count influences the cell culture methodology (plating, feeding, expansion, etc). Moreover, in the batch release, the cell count is a key point to verify the compliance of each batch with CTP clinical protocols according to GCP [21-25]. On these bases, our primary aim was to validate a disposable cell count method in a GMP condition, to be included in the Validation Master Plan (VMP) to be submitted to regulatory bodies, in order to obtain accreditation of our Cell Factory to produce CTPs. The cell count is a potency test, according to EP, which define the cell, dose during *ex-vivo* expansion and batch release. In a GMP setting, the cell counting using disposable devices is a very important starting point because: 1) overcomes the problem of chamber washing after use allowing cell count in a clean environment, such as in a Cell Factory; 2) allows a single use of the count chamber which can then be trashed; 3) avoids the disposal of toxic substance such as vital dye (e.g. Trypan Blue) or lysing solution (e.g. Tuerk solution). On these bases, among other disposable cell count chambers, we tested Fast Read 102 ® device as in our lab we have experience on cell counting by it. Firstly we tested whether Fast Read 102® slides might substitute the Bürker chamber in terms of accuracy. Once this hypothesis was proved, we tested repeatability, linearity and range only by using Fast Read 102 ®. Two cell subpopulations were chosen for the validation procedure: MNCs, as a subset of lymphocytes, and MSCs, both CTPs that we will produce for immunotherapy and regenerative medicine.

For each test (accuracy, precision, repeatability, linearity and range), previously defined, the Operators, according to ICH Q2, performed three cell counts, under the same operating conditions.

Three repetitions for each count proved sufficient for a statistical analysis and the CV and SD we obtained corroborate, in our opinion, our experimental approach.

As cell counting method with Fast Read 102® has proved to be accurate we decided to use the disposable Fast Read 102® chamber, instead of the Bürker chamber, for all subsequent tests.

Our data demonstrated that the Fast Read 102® count method is accurate, precise and ensures the linearity of the results obtained in a range of cell dilutions. According to acceptance criteria the total cell count CV% (*inter* and *intra* operator) was 10% and the viable cell count CV% (*inter* and *intra* operator) was <5%. According to ICHQ2, the cell count is a potency test, so the Detection Limit and Quantitation Limit are not required. However in the contest of linearity and range test we verified that, for all cell subset used, the optimal counting cell concentration range is between 1:8 and 1:128 dilutions. Moreover matching all our accuracy, precision and linearity range data, we can conclude that the minimal and the maximal cells which can be counted are about from 30,000 to 600,000 cells respectively.

Despite being a niche article, our paper might be very important for the scientific community in the cell therapy field because our data demonstrated that cell count, by Fast Read 102®, should satisfy Pharmacopoeia rules, and might be used, in clean rooms, to dose CTPs.

## Abbreviations

BM: Bone marrow; CBMPs: Cell based medical products; CTP: Cell therapy products; CV: Coefficient of variation; EC: European community; EU: European union; GCP: Good clinical practice; GMP: Good manufacturing practice; ICH Q2: International conference on harmonization Q2; MNC: Mononuclear cells; MSC: Mesenchymal stem cells; MCPK: MesenCult® Proliferation Kit; Op: Operator; PB: Peripheral blood; SD: Standard deviation; wBM: Whole bone marrow; wPB: Whole peripheral blood.

## Competing interest

The authors declare that they have no competing interests.

## Authors’ contributions

MG participated in the design of the study, carried out the experiment, acquired, analyzed, interpreted data, performed the statistical analysis, and drafted the article. SC participated in the design of the study, carried out the experiment, acquired, analyzed, interpreted data, performed the statistical analysis, and drafted the article. DR participated in the design of the study, interpreted data, and performed the statistical analysis. KM participated in the design of the study, interpreted data, and performed the statistical analysis. FS participated in the design of the study, interpreted data, and performed the statistical analysis. MM participated in the design of the study, interpreted data, and performed the statistical analysis. ES participated in the design of the study, interpreted data, and performed the statistical analysis. LC participated in the design of the study, interpreted data, and performed the statistical analysis. IF conceived the study, participated in the design of the study, interpreted data, and drafted the article. FF conceived the study, contributed reagents, materials, analysis tools, and interpreted data. All the authors critically revised the article for important intellectual content, and read and approved the final manuscript.

## Authors’ information

**MG:** PhD, Qualified Operator, Production

**SC:** PhD, Qualified Operator, Quality Control

**DR:** MSc, Head, Quality Control

**KM:** BSc, Head, Production

**FS:** DipBiol, Qualified Operator, Production

**MM:** PhD, Qualified Operator, Production

**ES:** PhD, Qualified Operator, Production

**LC:** PhD, Qualified Operator, Quality Control

**IF:** MSc, Qualified Person, Quality Assurance

**FF:** MD, Director
